# Potential role of passively increased muscle temperature on contractile function

**DOI:** 10.1007/s00421-022-04991-7

**Published:** 2022-06-30

**Authors:** Patrick Rodrigues, Gabriel S. Trajano, Ian B. Stewart, Geoffrey M. Minett

**Affiliations:** School of Exercise and Nutrition Sciences, Faculty of Health, Queensland University of Technology, A Wing O Block, Victoria Park Road, Kelvin Grove, Brisbane, QLD 4059 Australia

**Keywords:** Heat stress, Passive heating, Muscle temperature, Neuromuscular function, Muscle strength, Rate of force development, Muscle fluid, Calcium kinetics

## Abstract

Declines in muscle force, power, and contractile function can be observed in older adults, clinical populations, inactive individuals, and injured athletes. Passive heating exposure (e.g., hot baths, sauna, or heated garments) has been used for health purposes, including skeletal muscle treatment. An acute increase in muscle temperature by passive heating can increase the voluntary rate of force development and electrically evoked contraction properties (i.e., time to peak twitch torque, half-relation time, and electromechanical delay). The improvements in the rate of force development and evoked contraction assessments with increased muscle temperature after passive heating reveal peripheral mechanisms’ potential role in enhancing muscle contraction. This review aimed to summarise, discuss, and highlight the potential role of an acute passive heating stimulus on skeletal muscle cells to improve contractile function. These mechanisms include increased calcium kinetics (release/reuptake), calcium sensitivity, and increased intramuscular fluid.

## Introduction

Reduced ability to produce muscle force and power, and reduction in muscle mass, are common in elderly and clinical populations (e.g., people with cancer, AIDS, renal failure, sepsis, and diabetes) (Lecker et al. 2006; Pinel et al. 2021; Suetta et al. 2019). These decreases in neuromuscular activity impair functional capacity, mobility, and walking efficiency (Alexander 1996; Satariano et al. 2012). In addition, increased fall risk and reduced independent, healthy living (Orssatto et al. 2020; Pinel et al. 2021), leading to lower quality of life (Venturelli et al. 2018), are experienced. Moreover, bed rest due to illness and limb immobilization associated with musculoskeletal injury can also reduce neuromuscular function irrespective of age or health status. A short period of skeletal muscle unloading (10 days of bed rest) is sufficient to reduce muscle mass and force in healthy young adults (~ 23 years), with losses in contractile force occurring quicker than muscle atrophy (Monti et al. 2021).

Passive heating therapy, generally sauna and hot-water immersion, has long been used (460 BC) for health and medical purposes (Barfield and Hodder 1987; Papaioannou et al. 2016). The medical and health benefits of passive heating include the reduced effects of aging (e.g., improved vascular compliance and endothelial function; neurodegenerative and cardiovascular disease prevention) (Hunt et al. 2020; Patrick and Johnson 2021), extended healthspan (i.e., healthy longevity) (Patrick and Johnson 2021), and skeletal muscle treatment (e.g., promotes hypertrophy; slows atrophy; increases strength) (Goto et al. 2011; Hafen et al. 2019; Kim et al. 2020a).

Specific to the neuromuscular system, passive heating interventions increase maximal voluntary contraction (MVC) torque (11 days to 10 weeks) (Kim et al. 2020a; Goto et al. 2011; Racinais et al. 2017) and muscle mass (10 weeks) (Goto et al. 2011) and decreases muscle atrophy rate after 10 days of unloading (immobilized legs) (Hafen et al. 2019). These responses are potentially explained by the many physiological mechanisms triggered when muscles are exposed to heat stress, including the upregulation of heat shock and enzymatic proteins, especially heat shock protein 72, and a subsequent inflammatory cascade associated with muscle growth and activation of the thymoma viral proto-oncogene 1 and mammalian target of rapamycin (Akt/mTOR) pathway (see Rodrigues et al. (2020a) for systematic review). The chronic effects of passive heating therapy on skeletal muscle cells have been extensively discussed in a recent review by Kim et al. (2020b). Accordingly, this review focuses on the acute effects of passive heating on muscle contractile function.

Increased muscle contractile capacity occurs with higher muscle temperature after acute bouts of passive heating (lower body [Rodrigues et al. 2021]; whole body [Morrison et al. 2004; Périard et al. 2014; Racinais et al. 2017]). For example, an increased rate of force development (RFD) was observed after a passive increase in muscle temperature during voluntary (Rodrigues et al. 2021) and involuntary (electrically evoked twitch) contraction (Racinais et al. 2017; Rodrigues et al. 2021). The RFD is a relevant measure of neuromuscular function when the time available to produce force is limited (e.g., reversing a fall) (Gordon et al. 2018; Folland et al. 2014) and is associated with daily functional tasks (Maffiuletti et al. 2010). In contrast, acute studies involving systemic hyperthermia (i.e., increased core and muscle temperature) during passing heating exposure have reported decreases in MVC torque and voluntary activation level (measured by superimposed twitch) (Morrison et al. 2004; Périard et al. 2014; Racinais et al. 2017; Thomas et al. 2006; Todd et al. 2005). These decrements in MVC during hyperthermia align with declines in central neural drive to the working muscles (Racinais and Oksa 2010). Critically, voluntary muscle activation is impaired by increased core temperature rather than elevated local muscle temperature (Thomas et al. 2006), and MVC and voluntary activation return to initial levels once baseline core temperature is resumed (Morrison et al. 2004).

Interestingly, even with increased core temperature, evoked twitch assessments associated with rapid contraction properties reportedly improve with passive heating (i.e., increased RFD; decreased time to peak torque, half-relaxation time, and electromechanical delay) (Morrison et al. 2004; Périard et al. 2014; Racinais et al. 2017; Rodrigues et al. 2021; Thomas et al. 2006; Mornas et al. 2021), revealing the potential role of peripheral mechanisms in enhancing muscle contractile function. Increases in muscle contractile properties accompanied by elevated muscle temperature have been attributed to increases in calcium (Ca^2+^) influx and Ca^2+^ sensitivity (Kobayashi et al. 2005), adenosine triphosphate splitting (ATPase) activity (Rall and Woledge 1990), and increased intramuscular fluid (Blazevich and Babault 2019). Hence, this review aims to summarise, discuss, and highlight the potential role of an acute passive heating stimulus on skeletal muscle cells to improve contractile function.

## Muscle temperature and contractile function

Acute increases in muscle temperature achieved with passive heating improve muscle contractility (Close and Hoh 1968), strength (Davies and Young 1983), and power (Bergh and Ekblom 1979). Although using small sample sizes (5–10 participants), preliminary studies in humans observed improvements in muscle contractile function after passive heating (Binkhorst et al. 1977; Davies and Young 1983; Davies et al. 1982; Sargeant 1987). Increases in voluntary maximal power (Binkhorst et al. 1977; Sargeant 1987) and evoked muscle contraction (i.e., time to peak torque and half-relaxation time) (Davies and Young 1983; Davies et al. 1982) were found after a hot-water immersion session. Furthermore, recent studies (Mornas et al. 2021; Périard et al. 2014; Racinais et al. 2017; Rodrigues et al. 2021) confirmed the improvements in evoked contraction (i.e., RFD, time to peak torque, half-relaxation time, and electromechanical delay) with increased muscle temperature after passive heating. Notably, these changes examined via the evoked contraction technique point to specific peripheral mechanisms involved in the muscle contraction.

Although increases in muscle contractile function have been found after a passive increase in muscle temperature, MVC torque and peak twitch torque do not increase (Davies et al. 1982; Morrison et al. 2004; Périard et al. 2014; Racinais et al. 2017; Thomas et al. 2006). This suggests that increased muscle temperature is more effective on fast contraction force (e.g., RFD and time to peak torque). The peak twitch torque is associated with the number of interactions between actin and myosin; in contrast, time to peak torque and half-relaxation time represent the rate of Ca^2+^ release and reuptake (Ca^2+^ ATPase) from the sarcoplasmic reticulum (Fitts and Holloszy 1978; Stein et al. 1982). The release of the Ca^2+^ into the myoplasm results in the binding of Ca^2+^ to troponin C unblocking the sites between the actin and myosin heads (cross-bridges formation), subsequently producing force development. The Ca^2+^ reuptake to the sarcoplasmic reticulum leads to dissociation of Ca^2+^ from troponin C and subsequent muscle relaxation (Nielsen 2009). Therefore, rather than increasing the number of cross-bridges (simultaneously), it seems that passive heating can increase the rate of cross-bridge attachment and detachment. Moreover, increases in blood flow, and subsequently in muscle fluid, in response to passive heating may also increase the rate of cross-bridge formation (Edman and Andersson 1968) and the muscle shortening velocity (Edman and Hwang 1977), besides increasing muscle stiffness, causing a positive effect on RFD (voluntary and evoked contraction) (Eng et al. 2018).

In summary, although increases in MVC torque and peak twitch torque are not observed with a passive increase in muscle temperature, enhanced voluntary and involuntary fast force contraction properties are apparent. These improvements in muscle contractile function have been noticeably observed during electrically evoked contraction assessments, suggesting increases in cellular Ca^2+^ kinetics (Ca^2+^ release and reuptake) and muscle fluid after an acute session of passive heating.

### Passive heating and Ca^2+^ kinetics

Muscle contraction and relaxation temporal characteristics are shortened when the muscle temperature is raised (Close and Hoh 1968; Segal and Faulkner 1985; Ranatunga 1982). The relationship between increased muscle temperature and the force–time curve has been associated with increases in Ca^2+^ kinetics (release/reuptake) and myosin ATPase activity (Stein et al. 1982; Barany 1967). This section provides compelling evidence that changes in Ca^2+^ homeostasis triggered by a passive rise in muscle temperature play a vital role in increasing muscle contractile function.

Kobayashi et al. (2005) found greater muscle mass (in male rats) associated with increased calcineurin expression in both the slow-twitch and fast-twitch soleus muscle after one passive heating session. According to the authors, the increase in soleus muscle mass following passive heating was partially suppressed by the calcineurin inhibitor cyclosporine A. As elevations in intracellular Ca^2+^ activate calcineurin, this study was the first to propose that heat stress may trigger myoplasmic Ca^2+^ accumulation (Kobayashi et al. 2005). Activation of Ca^2+^ signalling from the sarcoplasmic reticulum through the transient receptor potential vanilloid 1 (Trpv1) channel has been suggested as a critical trigger for skeletal muscle hypertrophy and atrophy treatment (studies in myoblast cells) (Ito et al. 2013; Obi et al. 2019). Although the changes in muscle mass are not the central point of this review, recent studies have demonstrated that passive heating increases intracellular Ca^2+^ influx by increasing the myotube formation through Trpv1 channels in a temperature-dependent manner (Obi et al. 2017, 2019).

**Fig. 1:**
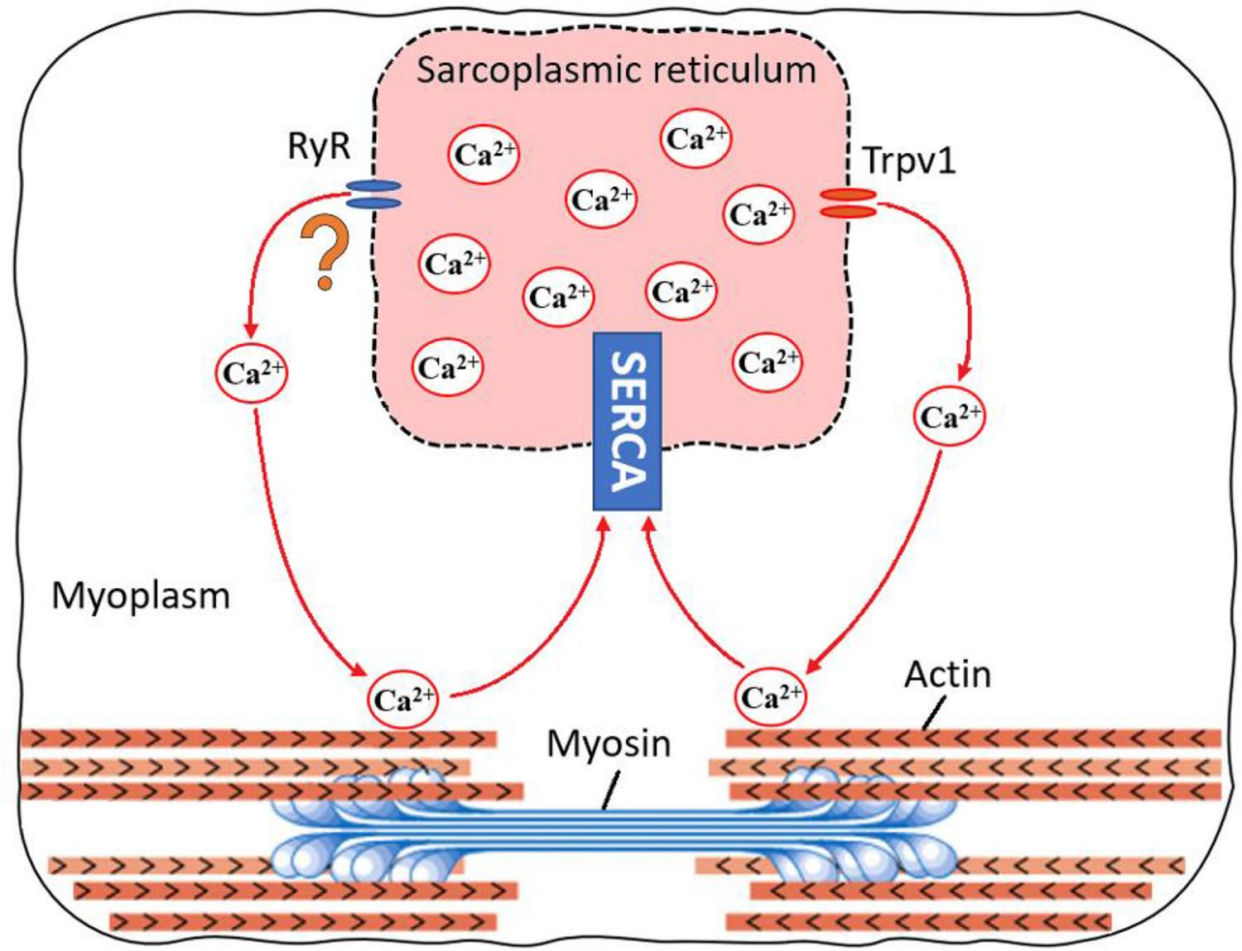
Conceptual diagram of increased Ca^2+^ kinetics (release/reuptake) triggered by a passive rise in muscle temperature. Arrows indicates the increased Ca^2+^ flow from sarcoplasmic reticulum trough ryanodine receptors (RyR) and transient receptor potential vanilloid 1 (Trpv1) channels to myoplasm. However, it is unclear if sarcoplasmic Ca^2+^ release is triggered by RyR and Trpv1 or Trpv1 alone. Myoplasm Ca^2+^ unblocks the sites between actin and myosin heads increasing the cross-bridge formations. Subsequently, the sarcoplasmic reticulum Ca^2+^ ATPase (SERCA) channel reuptakes the Ca^2+^ back to the sarcoplasmic reticulum

During muscle contraction, sarcoplasmic Ca^2+^ is also released via ryanodine receptors (RyR) in response to the sarcolemma depolarization. Changes in myoplasmic Ca^2+^ concentration have been tested by the low/high-frequency twitch ratio (Millet et al. 2011) once the RyR (voltage-sensitivity Ca^2+^ release channel) controls the force–frequency slope responses in the muscles (Nielsen 2009). However, more studies are needed to test the low/high-frequency twitch ratio after passive heating to reveal whether a passive rise in muscle temperature increases the release of Ca^2+^ via RyR channels. It is unclear whether passive heating affects RyR or the increase in myoplasmic Ca^2+^ concentration occurs via Trpv1 gates only. Nevertheless, decreases in twitch contraction half-relaxation time have been observed after passive heating (Davies and Young 1983; Davies et al. 1982; Rodrigues et al. 2021), suggesting increased Ca^2+^ reuptake back to the sarcoplasm by the sarcoplasmic reticulum Ca^2+^ ATPase (SERCA) channel (Fig. 1).

It remains unclear if passive heating would increase Ca^2+^ sensitivity (i.e., increase cross-bridge-generated muscle force for a given level of muscle or fibre activation). The affinity of the Ca^2+^ to troponin C decreases with increases in temperature as Ca^2+^ binding is an exothermic reaction (Potter et al. 1977). Alternatively, decreased ionic strength, induced via greater muscle fluid (explained in the next section), increases muscle fibre force and shortening velocity (Edman and Andersson 1968; Edman and Hwang 1977; Sugi et al. 2013, 2015). The effect of temperature on Ca^2+^ sensitivity appears modest and probably not a critical factor influencing cross-bridge formations when temperature rises (Blazevich and Babult 2019). However, increases in Ca^2+^ sensitivity after passive heating should not be disregarded. The increase in cross-bridge formation for a given muscle activation may occur after passive heating not by elevated temperature per se, but by consequences of passive heating exposure (i.e., increased intramuscular fluid).

In summary, one passive heating exposure seemingly increases muscle cell Ca^2+^ kinetics. It remains unclear if sarcoplasmic Ca^2+^ release is triggered by RyR and Trpv1 or Trpv1 alone. However, the increases in intracellular Ca^2+^ concentration found after passive heating may explain the improvements in twitch contraction RFD, time to peak torque, and electromechanical delay. Moreover, the Ca^2+^ reuptake via the SERCA channel explains the decrease in half-relaxation time.

## Passive heating and muscle fluid

**Fig. 2:**
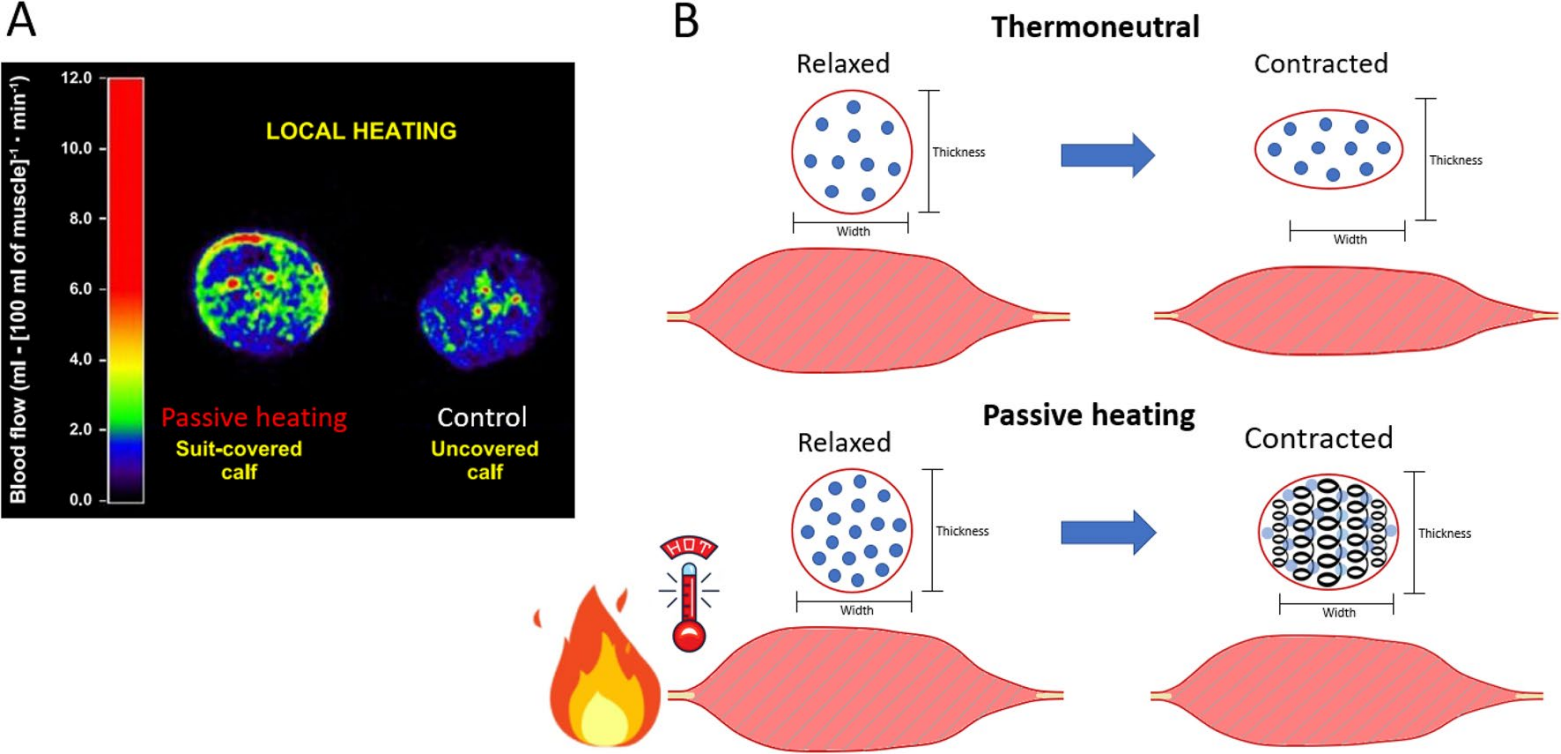
**a** Representative cross-sectional positron-emission tomography (PET) blood flow image from the middle calf at normothermic (control) condition (muscle temperature at ~ 33.4 °C) and immediately after a local passive heating session (muscle temperature at ~ 37.4 °C). Image taken with permission from Heinonen et al. (2011). **b** A hypothetical schematic representation of changes in muscle fibre shape during contraction at thermoneutral and passively heated condition. The *red circles* denote the cross-sectional area of the muscle fibre, and the *blue dots* denote the intramuscular fluid accumulation. In a thermoneutral condition, during contraction, the muscle fibre tends to compress in the thickness direction changing the shape of the muscle expanding in the radial direction (i.e., width) (Eng et al. 2018). After a passive heating session, with increased muscle temperature and fluid, the incompressible nature of the fluid inside the cells increases the muscle cells’ pressure, decreasing muscle fibre deformation during contraction and creating a spring-like property

Passive heating increases local peripheral tissue perfusion via thermosensitive mechanisms that increase microvascular blood flow, likely via heat-modulated rheology and/or vasodilation (Koch Esteves et al. 2021; Chiesa et al. 2015). Koch Esteves et al. (2021) found a strong relationship between increases in local temperature (skin and muscle temperature) and local blood flow (thigh: *r*^2^ = 0.89; leg: *r*^2^ = 0.99). Local muscle blood flow increases linearly with muscle temperature (Pearson et al. 2011) and can rise by 61% after heat exposure (Heinonen et al. 2011) (Fig. 2a). This section discusses important mechanisms of how increases in muscle fluid elicited by passive heating can enhance muscle contractile function.

Three potential mechanisms are suggested to increase muscle blood flow under external heat stress. First, increased muscle temperature increases vasodilation resistance vessels in human microvascular circulation (Heinonen et al. 2011; Koch Esteves et al. 2021). Second, a higher temperature increases oxygen consumption of the tissue (Chiesa et al. 2015; Pearson et al. 2011), which may initiate metabolically induced vasodilation (Heinonen et al. 2011). And, third, ATP release from red blood cells is a potent endothelium-dependent vasodilator and sympatholytic agent (Rosenmeier et al. 2004; Wood et al. 2009; Segal 2005; Ellsworth 2004). Pearson et al. (2011) observed increased arterial ATP plasma during passive heating. They found that arterial ATP plasma concentration has a strong correlation with muscle vascular conductance (*r*^2^ = 0.87) and muscle temperature (*r*^2^ = 0.85). Furthermore, increases in erythrocyte ATP release are sensitive to passive heating in isolated red blood cells, but not in other blood cells (Kalsi and González-Alonso 2012; Kalsi et al. 2017). This suggests that the temperature-dependent release of ATP from erythrocytes might be a critical mechanism regulating blood flow during local hyperthermia (Kalsi and González-Alonso 2012). Consequently, the acute rise in muscle temperature with passive heating seemingly plays an important role in increasing muscle blood flow.

Increases in muscle blood flow, and so in muscle fluid, may potentially augment muscle contractile function in two ways; by increasing cross-bridge formation and muscle stiffness. Water movement inside the cellular muscle space decreases muscle fibre hypotonicity (i.e., ionic strength) (Sjøgaard et al. 1985), which has been shown to increase muscle fibre force and fast contraction (Edman and Andersson 1968; Edman and Hwang 1977; Sugi et al. 2013, 2015). The magnitude of Ca^2+^-activated isometric force increases with decreasing ionic strength in skinned muscle fibres (Gordon et al. 1973; Thames et al. 1974). Nevertheless, some in vitro studies (Chalovich and Eisenberg 1982; Chalovich et al. 1981) suggest that myosin head binds to actin filaments (surrounded by the troponin–tropomyosin system) to the same extent independently of the presence of the Ca^2+^ at low ionic strength. Together, this evidence indicates that at low ionic strength, myosin heads and actin filaments increase cross-bridge formations in relaxed muscle fibres. Sugi et al. (2013) found approximately twofold increases of Ca^2+^-activated isometric force when ionic strength was decreased using potassium chloride (KC1). They also found a progressive increase in muscle fibre stiffness, while ionic strength decreased. Ca^2+^ activation increases force and stiffness almost concurrently when ionic strength decreases until reaching their respective maximal steady values (Sugi and Tsuchiya 1988; Sugi et al. 1992). Therefore, the influence of reduced ionic strength would subsequently increase the number of strongly bound, force-producing cross-bridge formations during muscle contraction, and perhaps, increase force due to muscle fibre elastic components. Regardless of the exact mechanism, reduced ionic strength seemingly increases cross-bridge formation and muscle contractile function. Theoretically, these responses should have a similar time course of change to muscle temperature and blood flow (Blazevich and Babault 2019).

The elastic structures of muscle fibre strongly influence time to peak torque and RFD (Josephson and Edman 1998; Edman and Josephson 2007). During muscle contraction, the fibres tend to compress in the direction of thickness, changing the muscle fibres’ shape and expanding radially when they shorten (Eng et al. 2018). Increased muscle fluid increases passive stiffness, which unloads the muscle during shortening (Edman and Andersson 1968). The incompressible nature of the fluid inside muscle cells decreases the rate of muscle fibre deformation during contraction. This is described as a spring-like property (Eng et al. 2018) (Fig. 2b). The muscle fluid’s greater incompressible characteristics increase the forces transmission during muscle contraction (Eng et al. 2018). Hence, the increases in muscle fluid caused by passive heating increase the internal muscle cell pressure (spring-like), directly affecting (increasing) muscle force and velocity. Moreover, intramuscular water accumulation might positively influence hydrogen-bonding effects (Zhang et al. 2007). This would increase muscle shortening velocity (i.e., increase the gear ratio) and increase muscle force by allowing less fibre shortening for a given muscle distance (Blazevich and Babault 2019). Therefore, fluid-dependent increases in stiffness optimize muscle force–velocity and force–length properties during contraction (Edman and Andersson 1968; Eng and Roberts 2018). Although an acute session of passive heating increases muscle blood flow (Pearson et al. 2011; Heinonen et al. 2011), it is unclear how much increase in the intracellular fluid it generates. Nevertheless, increased muscle microvascular circulation and intracellular fluid would increase the whole muscle–tendon unit stiffness. This will contribute to positively changing the elastic properties of the muscle and increasing force transmission during fast contractions (voluntary and involuntary).

In summary, passive heating of muscle temperature acutely increases muscle blood flow, triggering two critical effects on muscle cells: decreased ionic strength and increased internal pressure. The decrease in ionic strength increases cross-bridge formation, while greater intramuscular pressure increases muscle–tendon unit stiffness and force transmission. Intramuscular fluid may also contribute to cross-bridge formation (apart from ionic strength). These mechanisms are associated with evoked twitch contraction and voluntary rapid force production. Accordingly, these changes in muscle cells can explain the improvements in voluntary RFD, time to peak torque, and electromechanical delay assessments by increased muscle temperature observed after a single session of passive heating.

## Technical consideration

This review discusses the potential mechanisms to increase muscle contractile function when muscles are acutely exposed to passive heating. Some studies suggest that these mechanisms respond in a muscle temperature-dependent manner (Obi et al. 2016, 2019; Pearson et al. 2011; Sugi and Tsuchiya 1988; Sugi et al. 1992). However, increasing muscle temperature to extreme physiological levels may decrease muscle contractility (Van der Poel and Stephenson 2002). When muscle temperature is elevated to a certain level, the heat stimulates protein degradation more than protein synthesis (Baracos et al. 1984; Luo et al. 2000). Proteolysis and ultrastructural damage have been observed (in vitro) when muscle temperature exceeds 42 °C (Baracos et al. 1984; Essig et al. 1985). Thus, it seems that there is an optimal level of increased muscle temperature to improve skeletal muscle function. Manipulating muscle temperature within a range between 34 and 41 °C would be a reasonable way to achieve increased acute muscle contractile function via passive heating.

Hot-water immersion is a tolerable, feasible, and affordable way of passive heating for clinical and home prescription (Rodrigues et al. 2020b). The water temperature and duration of passive heating are crucial factors to be controlled when administrating hot-water immersion. Studies have applied hot-water immersion in upper and lower limbs for 20 to 90 min at water temperatures varying from 39 to 46 °C, reaching a muscle temperature of 37–40 °C (see Table 1). Of course, longer passive heating sessions and hotter water temperatures will achieve higher levels of muscle temperature. However, the water temperature will also depend on the patient’s tolerance to the heat and thermoregulatory responses.

**Table 1:** Summary of studies using hot-water immersion as a passive heating strategy

Authors	Participants	Body segment	Water temperature	Passive heating duration	Muscle temperature
Binkhorst et al. (1977)	10 healthy males (age not reported)	Single forearm	39 °C	30 min	~ 37.3 °C
De Ruiter et al. (1999)	15 healthy people: 21 ± 1 yrs (8 females and 7 males)	Single forearm	45 °C	20 min	~ 37 °C
Edwards et al. (1972)	10 healthy males: 25 ± 3.5 years	Single lower limb	44 °C	45 min	~ 38.6 °C
Davies and Young (1983)	5 healthy males: 22 ± 1 years	Single lower limb	46 °C	30–45 min	~ 40 °C
Sargeant (1987)	4 healthy people: ~ 27 years (1 female and 3 males)	Both lower limbs (gluteal fold level)	44 °C	45 min	~ 39 °C
Faulkner et al. (2017)	14 males divided into two groups 7 lean: 24 ± 3.5 years 7 overweight: 31 ± 12 yrs	Lower body (waist level)	40 °C	60 min	↑ ∆ 2.3 °C (baseline values not informed)
Rodrigues et al. (2020b)	15 healthy people: 25 ± 5.6 years (9 females and 6 males)	Lower body (waist level)	42 °C	90 min	~ 39 °C (↑ ∆ 2.8 °C)

Most of the articles using hot-water immersion as a passive heating strategy have studied young healthy males (Table 1), indicating a need for more studies on other populations (Hutchins et al. 2021). Men and women, for instance, have different thermoregulation responses under heat stress due to the female physiology (e.g., sex hormones, body water regulation, exercise capacity, and body mass, size, and composition) (Kaciuba-Uscilko and Grucza 2001). Older adults, particularly those with age-associated chronic health conditions (e.g., cardiovascular disease, hypertension, obesity, and type 2 diabetes), present impaired physiological thermoregulation (e.g., regulation of body temperature, hemodynamic stability, and hydration status) (Meade et al. 2020). Accordingly, more studies on women and older adults using hot-water immersion are required to test tolerability, safety, and thermoregulatory response for the passive heating prescription.

Notably, the studies in the table below have applied hot-water immersion to single limbs or the lower body, but care must be taken if hot-water immersion is applied to the whole body, especially in vulnerable older demographics. A smaller body surface area is available for heat dissipation during whole-body hot-water immersion, increasing core temperature and cardiovascular strain. Therefore, lower water temperature or passive heating duration should be considered for safety purposes. Although not informing muscle temperature, studies (Brunt et al. 2016, 2018) have applied whole-body hot-water immersion (up to the shoulders level) for 60 min at 40.5 °C in young healthy people (8 males, 12 females, ~ 22 years) with reasonable safety (core temperature between 38.5 and 39.0 °C).

## Conclusion and perspectives

This review offers insights into increasing muscle contractile function after a passive heating exposure in response to increased muscle temperature. An acute passive increase in muscle temperature enhances voluntary and involuntary RFD, time to peak torque, half-relaxation time, and electromechanical delay. The potential mechanisms involved in these changes are muscle temperature, increased Ca^2+^ kinetics, and muscle fluid. These responses to passive heating may help people with low muscle contractile activity, including older adults, physically inactive individuals, and clinical populations. Moreover, passive heating could help active people or athletes temporarily unable to exercise. The early loss of muscle force induced by inactivity is predominantly caused by maladaptive changes in the neuromuscular system rather than decreases in muscle mass. Monti et al. (2021) submitted ten young, healthy people (~ 23 years) to 10 continuous days of supervised bed rest in a hospital room. They concluded that the increased neuromuscular junction instability and impaired intracellular Ca^2+^ kinetics involved in the excitation–contraction coupling are the major determinants of declines in muscle force. Accordingly, there may be a role for passive heating in aiding physical capabilities in diverse scenarios (e.g., injury recovery, fall prevention interventions, and exercise mimetic strategies) to improve the quality of life and independence. Passive heating is an emerging and promising area of health treatment, and the effects of passive heating on muscle contractile function are an important subject for further research.

## Abbreviations

°C: Celsius degrees
Akt/mTOR: Activation of the thymoma viral proto-oncogene 1 and mammalian target of rapamycin
ATP: Adenosine triphosphate
ATPase: Adenosine triphosphate splitting
BC: Before Christ
Ca^2+^: Calcium
KC1: Potassium chloride
MVC: Maximal voluntary contraction
RFD: Rate of force development
RyR: Ryanodine receptors
SERCA: Sarcoplasmic reticulum Ca^2+^ ATPase
Trpv1: Transient receptor potential vanilloid 1
